# The Importance of Glycans of Viral and Host Proteins in Enveloped Virus Infection

**DOI:** 10.3389/fimmu.2021.638573

**Published:** 2021-04-29

**Authors:** Yuqing Li, Dongqi Liu, Yating Wang, Wenquan Su, Gang Liu, Weijie Dong

**Affiliations:** ^1^ Department of Biochemistry and Molecular Biology, Institute of Glycobiology, Dalian Medical University, Dalian, China; ^2^ The Queen’s University of Belfast Joint College, China Medical University, Shenyang, China; ^3^ Dalian Medical University, Dalian, China

**Keywords:** enveloped virus, glycan, host, immune escape, vaccine

## Abstract

Animal viruses are parasites of animal cells that have characteristics such as heredity and replication. Viruses can be divided into non-enveloped and enveloped viruses if a lipid bilayer membrane surrounds them or not. All the membrane proteins of enveloped viruses that function in attachment to target cells or membrane fusion are modified by glycosylation. Glycosylation is one of the most common post-translational modifications of proteins and plays an important role in many biological behaviors, such as protein folding and stabilization, virus attachment to target cell receptors and inhibition of antibody neutralization. Glycans of the host receptors can also regulate the attachment of the viruses and then influence the virus entry. With the development of glycosylation research technology, the research and development of novel virus vaccines and antiviral drugs based on glycan have received increasing attention. Here, we review the effects of host glycans and viral proteins on biological behaviors of viruses, and the opportunities for prevention and treatment of viral infectious diseases.

## Introduction

Animal viruses are parasites of animal cells that have characteristics such as heredity and replication, and are mainly composed of DNA or RNA, proteins and in some, a lipid membrane with glycoproteins (1). Commonly, viruses achieve invasion and infection with the help of the synthetic machinery of host cells. Viruses can be divided into two groups depending on whether they have a lipid bilayer membrane on their outer surface or not: enveloped viruses and non-enveloped viruses. Enveloped viruses have a lipid bilayer that comes from the host cell. It incorporates receptor attachment proteins and membrane fusion proteins that are both encoded by the virus. And all attachment or fusion proteins of enveloped viruses are modified by glycosylation. Glycosylation is important for the life cycle of the virus and plays essential roles in stability, antigenicity and infectivity of viruses (2). We summarize the functions of common enveloped viral glycoproteins in **Table 1**.

**Table 1:** Glycosylation of viral envelope proteins and its functions.

Virus	Viral glycoprotein^a)^	*N*-glycosylation sites	Viral glycoprotein functions
**HIV-1**	gp120	20~30 (3, 4)	Attachment, Transmission, Glycan shield
**H1N1**	HA	0~11 (5, 6)	Attachment, Glycan shield
**EBOV**	GP	11~18 (7)	Infectivity, Attachment, Glycan shield
**HCV**	E1, E2	4~15 (8)	Infectivity, Entry
**RABV**	GP	2 (9)	Infectivity, Entry, Virulence
**WNV**	prM, E	1~2 (10)	Entry, Release
**SARS-CoV-2**	S	22 (11)	Assembly, Attachment, Entry

^a)^These viral glycoprotein are classified as: (1) GP, glycoprotein; (2) HA, hemagglutinin; (3) E, enveloped proteins; (4) prM, premembrane; (5) S, spike protein.

**Figure 1:**
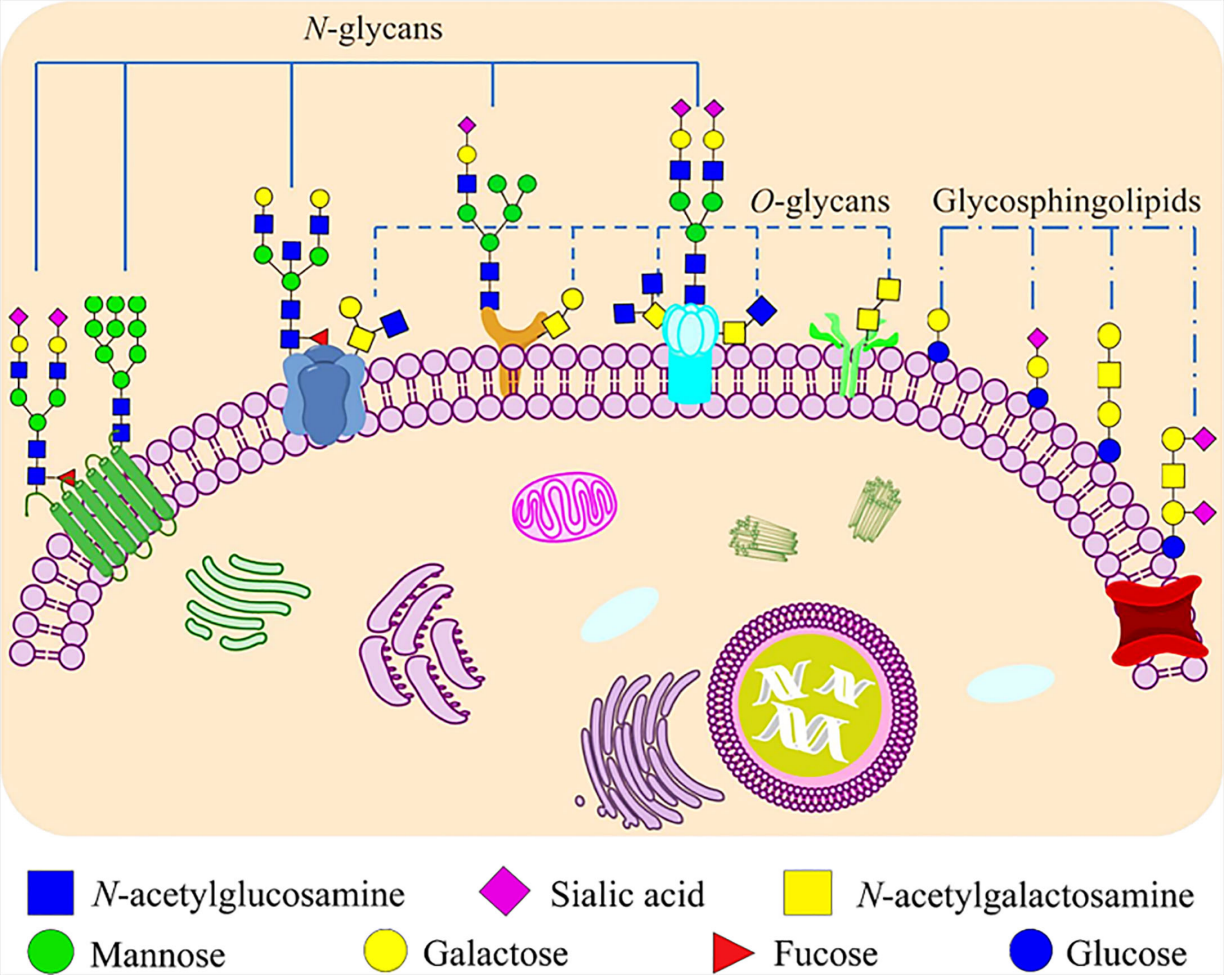
Glycoconjugates that formed by carbohydrates are covalently bonded to proteins and lipids on mammalian cell membranes. Proteins can be glycosylated and covalently bound to a polypeptide chain *via* an *N*-glycoside linkage to Asn or *via* an *O*-glycoside linkage to Ser/Thr. *N*-linked glycans share a common pentasaccharide core structure, which is composed of two GlcNAc and three mannoses. The main type of *O*-linked glycosylation is the mucin-type O-glycosylation, which has *N*-acetylgalactosamine (GalNAc) as a common core. Glycosphingolipids are ubiquitous molecules that formed *via* the covalent linkage between a glycan moiety and a ceramide.

Glycosylation is one of the most important post-translational modifications of proteins, and there are two main types: *N*-glycosylation and *O*-glycosylation (**Figure 1**). *N*-glycosylation means that *N*-acetylglucosamine (GlcNAc) in an oligosaccharide binds covalently to the polypeptide chain by an *N*-glycoside linkage with the amide nitrogen of an asparagine residue in the sequence Asn-X-Ser/Thr (X is any amino acid other than proline). The main type of *O*-glycosylation is the mucin-type *O*-glycosylation, which means that *N*-acetylgalactosamine (GalNAc) bonds covalently to the oxygen atom of the hydroxyl group of a serine or threonine residues replacing the hydrogen in the hydroxyl group to form an *O*-ligand glycoprotein by *O*-glycoside linkage. Other types of *O*-ligand subclasses also exist in animal cells, such as *O*-mannose glycosylation (*O*-Man), *O*-fucosylation (*O*-Fuc), *O*-galactose (*O*-Gal) and *O*-linked β-*N*-acetylglucosamine (*O*-GlcNAc) glycans (12). The formation and extension of glycans requires the synergistic completion of two types of glycan processing enzymes, one is glycosyltransferase which catalyzes the formation of glycoside linkages, and another is glycosidase which catalyzes the hydrolysis of glycoside linkages (13).

The entry of a virus into a host cell is closely related to the glycans on its own structural proteins. The glycoproteins of some viruses play an important role in host infection, especially in the recognition of the host cell and the interaction with other molecules in the cell after the attachment (14). Furthermore, due to the fact that viruses can fully depend on cellular host cells for their reproduction, thus, they must complete glycosylation with the help of host glycoprotein synthesis mechanism (1, 15). Viral glycoproteins are involved in many important biological processes, such as protein folding and stabilization, viral infection and invasion, recognition of host receptors and immune escape of the virus from the immune system (16). With the development of glycan-related research technology, the research and development of novel virus vaccines and antiviral drugs modified by glycosylation are receiving increasing attention. In this review, we will explore the effects of glycosylation of host and virus proteins on virus biological behavior, with the aim of providing a reference for the prevention and treatment of viral infectious diseases.

## Glycans of Viral Proteins and Their Functions

### Glycan Influences the Virus Replication Cycle

**Figure 2:**
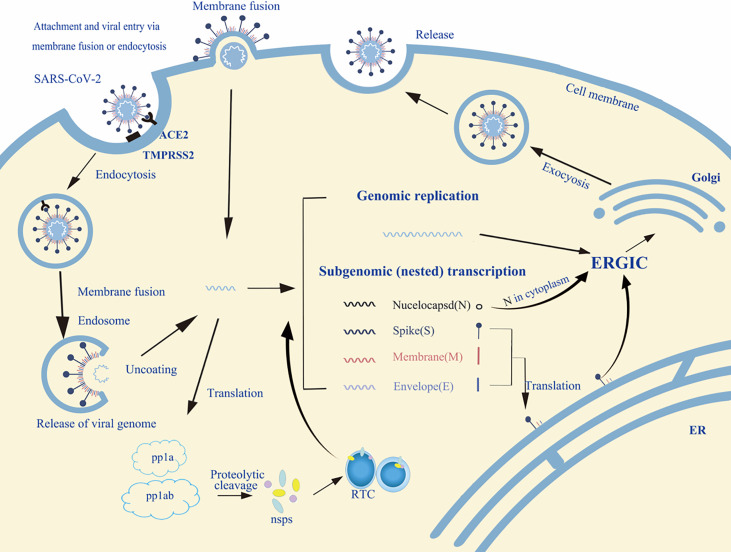
An overview of the life cycle of SARS-CoV-2 in host cells. Spike protein of SARS-CoV-2 binds to the host receptor ACE2 (angiotensin-converting enzyme 2); host factor TMPRSS2 (a cell surface serine protease enzyme), which helps the virion enter the host cells. SARS-CoV-2 enters through membrane fusion or endocytosis. Then it releases RNA to the cytoplasm. Some genomic RNA can be translated into viral proteins as the template, some of these proteins form a replication complex to make more RNA. Viral proteins and genome RNA are subsequently assembled into a new virion in ER and Golgi. Finally, the mature virions are released from the infected cell *via* exocytosis. The detailed life cycle of SARS-COV2 in host cells is referred to in the following literature (17–19). nsps, non-structural proteins; RTC, replication/transcription complex; ER, endoplasmic reticulum; ERGIC, ER-to-Golgi intermediate compartment.

The life cycle of enveloped viruses includes the processes of adsorption, penetration, uncoating, biosynthesis, assembly and release. **Figure 2** summarizes the life cycle of SARS-CoV-2. Viral replication depends on successful infection of the host cells. The replication cycle starts with a virus particle attaching to a specific receptor on the surface of a host cell, and viral entry can be realized by endocytosis (non-enveloped or enveloped viruses), membrane fusion (enveloped viruses) or direct fusion with the plasma membrane (20, 21). After internalization, the capsid is released into the cytoplasm with negative strand viruses, it is uncoating the nucleocapsid, which will be copied to produce the antigenome which itself is then used to transcribe many copies of the genome, or mRNA that are translated into viral proteins (17, 18). The viral glycopeptides are translated on the endoplasmic reticulum (ER) where the *N*-linked glycans are added and transported through the Golgi complex where the *N*-linked glycans are modified and the *O*-glycans are added (22, 23). At the same time, the viral genome with its associated proteins are transported to the Golgi apparatus, where they are released outside the cell by exocytosis after maturation (24). Glycoproteins are essential for the infectivity of the virions that have them.

#### Glycan Participates in Mutual Recognition Between Viruses and Host Receptors

The glycoproteins in the membrane of enveloped viruses bind specifically to the receptors on the cell membrane and cause membrane fusion enabling virus contents to enter the cell. In this process, the surface glycans of the virus can be involved in initiating the recognition of host cells and thus affect the organs and the cells that in the organs. Human immunodeficiency virus 1 (HIV-1) is an enveloped virus, and its envelope contains of gp120 surface protein and gp41 transmembrane protein. Moreover, gp120 is one of the most highly glycosylated proteins in nature. It accounts for approximately 50% of the total mass (25). These glycans not only affect the conformation of the envelope but also affect the entry and infectivity of the virus. It is generally believed that HIV-1 gp120 promotes viral entry by sequentially binding to CD4 and chemokine receptors CCR5 or CXCR4 on target cells (26). However, there have been many reports that gp120 can bind to various cell types independently of CD4. Some HIV-1 isolates were able to infect CD4-negative but CCR5 expressing cells due to the deficiency of Asn^197^ in gp120, which leads to the exposure of the CCR5-binding region of gp120, thus HIV-1 can enter cells (27). Similarly, the binding of virus to CD4 was reduced significantly by removing the glycan chains from gp120 by endoglycosidase treatment or *N*-glycosylation site mutation (28, 29). In contrast, in Vero cells, removing the *N*-glycan near the highly conserved receptor binding domain on the envelope glycoprotein (GP) of the Ebola virus (EBOV) increased GP-mediated virus entry efficiency (30).

Hemagglutinin (HA) and neuraminidase (NA) are the surface glycoproteins of influenza A virus. Influenza virus attaches to the surface of a host cell when its HA protein interacts with the terminal sialic acid (SA) of the host cell surface glycoproteins or glycolipids. On the other hand, NA can cleave SA residues from glycoproteins of the enveloped virus itself and enhance infectivity by preventing aggregation of virus particles (31). NA may also act on the SA residues of host mucin to gain access to the epithelial cells, playing a secondary role in helping viruses to enter host cells (32).

#### Glycan Affects the Folding and Transport of Viral Glycoproteins

One of the key roles of protein glycosylation is its effect on folding, structure, transport, and stability (33). Glycans can be structurally integrated into protein folding, and the interaction between the glycan and protein could stabilize the protein. Besides, glycans can also assist glycoprotein folding in the biosynthetic process by mediating interactions with chaperones (34). Calnexin (CNX) and calreticulin (CRT) act as chaperones to facilitate the correct folding of viral proteins. In cells, when all nascent *N*-glycans added to a protein, they have three terminal glucose residues. These residues will be removed sequentially, with α -glucosidase I (αGI) removing the outermost glucose residue and α-glucosidase II (αGII) removing the next two residues produce the immature GlcMan9GlcNAc2 *N*-glycans (35). CNX and CRT have a lectin domain, and this domain can specifically bind to immature GlcMan_9_GlcNAc_2_ *N*-glycans on misfolded proteins and recruit ATP, calcium, and protein disulfide isomerase A3 to promote the folding of glycoproteins, thereby regulating glycoprotein entry into the CNX/CRT folding cycle (36, 37).

Glycans on enveloped virus surface proteins can not only facilitate the folding of proteins but can also affect their transport. During HIV-1 replication, a high mannose type gp160 trimer assembles in the rough ER of host cells, and then gp160 is transported to the Golgi apparatus and cleaved by a furin-like protease in the late Golgi to its mature gp120 and gp41 proteins which remain associated (38, 39). HIV-1 gp41 contains four *N*-glycosylation sites, removing of Asn^332^ in gp41 can disrupt the proteolytic processing and the transportation of gpl60 (40, 41). Herpes simplex viruses 1 (HSV-1) and herpes simplex viruses 2 (HSV-2) are globally prevalent pathogens, which often lead to recurrent oral and genital ulcers (42). HSV encodes at least 12 different glycoproteins, and at least four of them are necessary and sufficient to mediate membrane fusion when they infect target cells, namely glycoprotein B (gB), gD, gH and gL (43–45). Similarly, mutations of N390, N483 or N668 in total seven potential *N*-glycosylation sites on gB of HSV-2 can reduce the ability of cell-cell fusion and virus entry. However, the mutation of N133 mainly prevents the transport of gB from the ER to the Golgi, thus affecting protein expression and the production of infectious virions (46).

#### Glycan Affects the Release of the Virions

Enveloped viruses mainly release their progeny by budding. Their envelope is derived from the host cell membrane, and the glycan on viral proteins can affect the release of progeny virions. For example, gC of HSV-1 mediates the attachment of HSV-1 to susceptible host cells by interacting with glycosaminoglycan (GAG) on the cell surface. Also, gC contains a mucin-like domain (MLD) located near the GAG binding site, which may affect the binding activity between the virus and GAG. HSV-1 mutants that lack these MLDs in gC and found that compared with natural HSV-1, the binding affinity of virions to the cell was reduced and the release ability of mutant virus particles from the surface of infected cells was also reduced (47). Similarly, glycosylation sites on the premembrane protein and enveloped proteins of West Nile virus (WNV) are cell type-dependent or even species-dependent and affect the release of virus and infection efficiency (10).

Moreover, some viruses have even evolved glycosidases to promote virus release (48). The most obvious example is that influenza virus NA cleaves SA residues from the surface of host cells, thereby reducing and weakening the ability of HA to bind to host cell glycoprotein receptors (49, 50). During the process of virus budding from the cell plasma membrane, HA proteins continue to bind virions to SA residues on the cell surface until the NA sialidase activity removes terminal SA residues.

### Glycan Affects Virus Transmission

During the process of viral evolution, viruses develop different glycosylation modifications, and *N*-glycosylation sites of proteins are added or deleted, these alterations can have an appreciable impact on the survival and transmissibility of a virus (16). For example, adding an *N*-glycosylation site to the HA protein can increase the sensitivity of the respiratory system to innate immune protein production, and reduce the transmissibility of influenza A viruses (51–53). Similarly, *N*-glycan of HIV gp120 is also of vital importance to viral infection and transmission (54). The majority of HIV *N*-glycan deficient mutants show decreased infectivity and transmission efficiency (N156Q, N197Q, N332Q, N386Q), but two of the *N*-glycan mutants (N230Q and N295Q) show increased infectivity and transmission efficiency (54). SARS-CoV-2 is currently causing a health crisis, the magnitude of which is rare in humans. The SARS-CoV-2 virus spike (S) glycoprotein is highly glycosylated, if the glycosylation sites of both N331 and N343 are mutated at the same time, the infectivity of SARS-CoV-2 can be significantly reduced, suggesting that their glycans are important for viral infectivity (55).

In addition, replication of the same virus in different cells can generate different glycosylation, which severely affects the transmission ability of the virus (56). For example, HIV from different cell lines has different glycosylation in its envelope proteins, and the glycosylation difference affects its interaction with Dendritic cell-specific ICAM-3 grabbing nonintegrin (DC-SIGN). HIV from T cell lines or peripheral blood mononuclear cells was well bound and transmitted by DC-SIGN, whereas HIV from macrophages was poorly bound and transmitted (57, 58).

### Glycan Affects Immune Escape

Enveloped viruses, such as HIV-1, influenza virus, SARS, and SARS-CoV-2 are a great threat to humans. The envelope proteins of these viruses are heavily glycosylated, and these glycans can hide an antigenic epitope to thereby avoiding recognition by neutralizing antibodies (nAbs) against that site and providing a convenient way for viruses to infect host cells and to promote immune escape. There are two main mechanisms by which viruses can escape the nAbs response.

#### Glycan Shielding

Despite major efforts to produce a vaccine for HIV-1, it is unfortunate that all of the traditional methods of vaccine preparation have generated little expected effect due to the great diversity of HIV-1 strains (59). Only a minority of people produce nAbs after receiving HIV vaccines. Mutation of Asn^332^ in gp120 after HIV infection and also nAbs that target this site were detected in the sera from two people who had responded to an HIV vaccine (a recombinant glycoprotein 120 vaccine), suggesting that HIV antigenic sites are blocked by glycans, resulting in most people failing to produce broadly nAbs (60). Similarly, patients with acute HIV infection do not produce detectable nAbs until at least 52 days after infection. Hardly any mutations were detected in the envelope proteins of the viruses that successfully escaped after nAbs suppression, while an increase in *N*-glycosylation was detected (61). Moreover, one of the two *N*-glycosylation sites of Ebola virus GP2 is mutated, which is very adverse to the antigenicity and immunogenicity of GP (62). All of these observations illustrate that highly glycosylated modifications can shield the antigenic sites of the virus and present challenges to antiviral therapy.

#### Antigenic Drift

Antigenic drift is a small variation in antigens caused by mutations in the genome, without new subtypes generation, but it often promotes immune escape and leads to the greater scope of the spreading, which often occurs in influenza viruses. H1N1 broke out in 1918/1919, infecting nearly half of the world’s population, with a mortality rate of 2.5%~5%, at least 50 million people died (63). Ninety-one years later, in April 2009, the new H1N1 virus appeared and spread rapidly around the world, causing an estimated 280,000 deaths worldwide. Low immunity of the population to the novel H1N1 strain was the main reason that led to its mass epidemic. HA is an effective target for nAbs, and mutations in the antigenic sites in its globular head region promote the immune escape of the virus. A complete analysis of the amino acid sequence of the 1918 and 2009 H1N1 pandemic viruses showed that the HA of these viruses can be glycosylated at the conserved glycosylation sites (64, 65). It was reported that highly glycosylated seasonal Influenza A viruses are inactivated by soluble lectins of the innate immune system (66). Compared with seasonal H1N1 and H3N2 influenza viruses, the HA globular regions of epidemic H1N1 are often modified by a low degree of glycosylation, which correlates with a greater difference at the amino acid level seen at or near the known antigenic sites located in the globular head of the HA (67, 68). Furthermore, that only influenza (H1N1) shows antigenic drift compared to several other RNA viruses that infect the respiratory tract (69). These antigenic drifts contribute to the immune escape of H1N1, contributing in part to the flu vaccine failing to provide protection (70).

### Glycan Affects Virulence/Pathogenicity of the Virus

There are many glycans in virus surface glycoproteins, and they affect the virulence of the virus by regulating the binding of the virus to host receptors, covering up antigenic sites, or stimulating host immune responses to affect virulence (71). These glycans play different roles in the virulence of the virus.

Rabies viruses (RABVs) are non-segmented, negative-stranded RNA viruses that belong to the genus *Lyssavirus* in the family *Rhabdoviridae*. The RABV genome encodes five structural proteins: nucleoprotein, phosphoprotein, matrix protein, GP, and large transcriptase protein (9). GP is the only viral transmembrane protein that is exposed on the virion surface that interacts and the target cell receptor. The GP of most RABVs has two *N*-glycosylation sites, Asn^37^ and Asn^319^. The *N*-glycan at Asn^37^ plays a role in promoting the propagation of the virus but also reduces the pathogenicity of the virus (53). Similarly, the addition of a single *N*-glycan at Asn^194^ or Asn^247^ also reduced the pathogenicity of street rabies viruses, confirmed by peripheral infection in mice (72). Furthermore, the virulence of H1N1 for mice decreased with an increase in the number of HA glycosylation sites (64).

On the other hand, the glycans of viral proteins can also enhance their virulence. For example, glycosylated and non-glycosylated E proteins of WNV are neurovirulent. However, viruses containing glycosylated E proteins are more neuroinvasive in BALB/c mice (73). Otherwise, the adding *N*-glycosylation of amino acids at either 154 or 156 increase neuro-invasiveness in mice, avian virulence, and vector competence (74).

## Host Glycans Affect Viral Infection

The specific binding of a virus to host receptor is a prerequisite for viral infection, and the viral glycans responsible for attachment and cell receptors of viruses play a decisive role. Many studies have focused on the functions of glycans on the viral protein: such as host cell recognition, replication, infection and immune escape. However, hosts and viruses have coevolutionary relationships, thus the functions of host receptors are also crucial, and all viruses must interact with the specific receptors when they enter a cell. The specificity of host receptors is considered to be one of the major factors in determining host range and tissue tropism. Host receptor refers to a component of the host cell membrane that can bind specifically to the virus, mediate virus entry, and promote virus infection. Its chemical nature is a glycoprotein, proteoglycan, lipid, or glycolipid (75, 76). According to their different functions, virus receptors can be divided into two categories: attachment factors and entry receptors (77). Entry receptors can bind to the virus and mediate the internalization of the virus, for example, HIV can bind to CD4. Attachment factors concentrate viruses on the cell surface and play an auxiliary role in the process of virus infection (78). For example, heparin sulfate, which is proteoglycan widely found on various cell surfaces and extracellular matrix in the body, can act as an auxiliary receptor of SARS-CoV-2 to promote the entry of virus (79). Therefore, glycans on a host cell receptor can play an important role in influencing viral infection as a functional receptor or attachment factor for the virus.

### Diversification of SA as Virus Receptor

**Figure 3:**
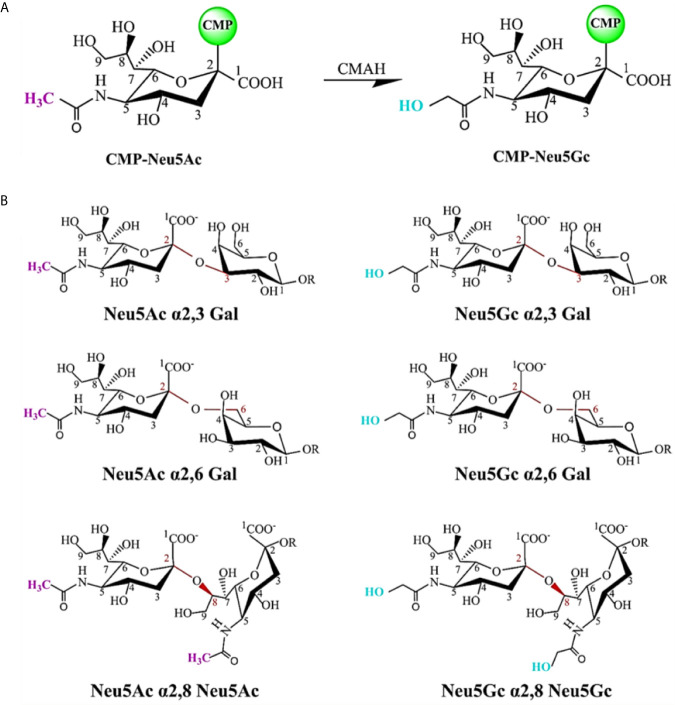
Chemical structure of sialic acid (SA) and glycosidic linkage types. **(A)** Neu5Ac and Neu5Gc are the most common two SAs. The C5 carbon is modified with an *N*-acetyl group to form Neu5Ac. CMP-Neu5Ac can be hydroxylated to CMP-Neu5Gc, catalyzed by cytidine monophosphate *N*-acetylneuraminic acid hydroxylase (CMAH). Most mammalian tissues contain both SAs. In contrast, this enzyme is inactive and Neu5Gc is not expressed in normal human tissues. **(B)** SAs attached to the terminal positions of *N*-glycans or *O*-glycans of glycoproteins and glycolipids *via* different glycosidic linkages as viral receptors. SA can be linked through an α2,3-linkage or an α2,6-linkage to a penultimate galactose residue; through an α2,6-linkage to *N*-acetylgalactosamine (GalNAc) moiety, and an α2,8-linkage to another SA moiety on a glycan.

SA is a naturally existing nine-carbon monosaccharide and has been identified as a functional receptor attached to the termini of *N*-glycans or *O*-glycans of glycoproteins and glycolipids (80). SA is one of the first sites for the contact between many pathogens and hosts because it presents on the outer surface of cells and mucosal tissues. SA carried by most mammals is called *N*-glycolylneuraminic acid (Neu5Gc) (**Figure 3A**); however, our ancestors also evolved another kind of SA, called *N*-acetylneuraminic acid (Neu5Ac), probably to resist the malaria parasite that was able to use Neu5Gc to enter the human body at that time. After completing this evolution, making some diseases seem more particularly specific to humans, such as typhoid, cholera, mumps, pertussis, asthma, and coronavirus diseases (COVID-19) (81). When SARS-CoV-2 enters the human body, it recognizes SA first, and search for its receptor at the same time, then it binds to angiotensin-converting enzyme 2 (ACE2), opening the access into the cell (82, 83).

Usually, SA exists as a bound sugar at the terminal positions of glycans *via* different glycosidic linkages (α2,3, α2,6, and α2,8). **Figure 3B** shows common SA linkage types. SA can be found in almost all animals, but the virus cannot infect all animals that have SA. Besides, different viruses infect different hosts by employing different receptors, which may be determined by the diversification of SA residue linkage types (**Figure 3B**), that is, viruses have a specific selectivity in the process of infecting the host. For example, the influenza virus binds to the receptor by recognizing SA residue at the terminal position of the receptor glycoprotein glycan chain, but it has the preference for certain types of SA (such as Neu5Gc and Neu5Ac). The preferential binding property may be attributed to the changes in the penultimate galactose residue linkage to SA. Avian influenza virus mainly binds to α2,3-linked SA residue, while human influenza virus preferentially binds to α2,6-linked SA residue (84, 85).

### Other Glycans of Host Cells Can Also Serve as Receptors Affect Viral Infection

Other glycans can also act as viral receptors to influence the entry of viruses, such as heparan sulfate, which can act as an initial adhesion receptors for various viruses, so it can help the virus adhere to the cells and mediate entry before the virus binds to the high-affinity receptors. HSV entry begins with the attachment of the virus to target cells through binding of HSV gC and/or gB to heparan sulfate proteoglycan syndecan-1 or syndecan-2 of the epithelial cell surface. Then, *via* various cell surface receptors such as nectin-1 or nectin-2. Finally, the attached virus begins to enter the cells (86–88). Like HSV-1, hepatitis C virus (HCV) enters cells by interacting with syndecan-1 or syndecan-4 on the surface of human hepatocytes to initiate its life cycle (89, 90). As for HCV, its functional receptors include SRB1, CD81, CLDN1, and OCLN, among which CD81 is the most important (91).

Also, neutral glycans may act as virus receptors, such as histo-blood group antigens, which are present in red blood cells, epithelial cells, and mucosal secretions, and play an important role as attachment factors for Rotavirus and Norovirus (92, 93).

## Development of Vaccine

Faced with the threat of new viruses, the effective prevention measure that we can take is to control the source of infection, cut off the main route of transmission and protect the susceptible population, these measures will be essential to bring the situation under control and to alleviate the negative effects of the epidemic. The definitive solution is effective vaccines that induce uninfected people to generate protective antibody and build long-term immune memory to combat the virus if the vaccine ever comes in contact with that virus at a later time.

### HIV

Although we know that the envelope proteins in HIV are the only target for nAbs (94), it is unfortunate that almost all of the traditional methods of vaccine preparation have generated little expected effect due to the great diversity of HIV-1 strains. Removal of the *N*-glycan modifications in the highly variable V1-V3 region of HIV-1 envelope protein improves the sensitivity of the virus to nAbs (95). Similarly, the gp120 complex mutated at the glycosylation site N448E can activate antiviral immunity better than the gp120 wild type. This suggests that removing *N*-glycans can increase the effectiveness of HIV vaccine (3). However, the heterogeneity of the HIV envelope, viruses tend to escape from most neutralizing antibody responses. Currently, HIV vaccine development research is focused on inducing unique broadly nAbs to act on diverse strains of HIV-1.

### Influenza Virus

Because of the antigenic drift of influenza viruses, current influenza vaccines need to be updated annually. The number and length of glycans on HA can affect the immune response; reducing the length of glycans can induce nAbs to make a stronger immune response to antigenic epitopes (96). The binding of nAbs to the HA glycans was inhibited at low temperature because the low temperature can stiffen the glycan structure (97). Interestingly, increased density of viral glycans in oligosaccharide-modified influenza viruses can directly activate the immune response and there exists a class of conserved epitopes in influenza viruses, that after adding hyperglycosylated artificially will become dominant (It was verified by mice *in vivo* experiment) (98). This finding suggests that further study on glycosylation of influenza virus HA protein can be helpful to develop antiviral drugs.

### HSV

Experimental vaccines against HSV-1 target particular viral glycoproteins. HSV envelope gD is expressed on the surface of the virus and induces a nAbs response. The largest current clinical trial of HSV subunit vaccines has found an inhibitory effect on HSV-1, but no effect against HSV-2 (99). A different attenuated vaccine strategy has been attempted by using HSV without glycoprotein D2, this vaccine can induce neutralizing antibody which displays antibody-dependent cell-mediated cytotoxicity activity to achieve better protection from HSV infection (100, 101).

### EBOV

The GP of EBOV is the main target of nAbs. Most of the current research on Ebola vaccines uses GP as the immunogen. Mature GP is composed of GP1 and GP2. They are presented on the surface of virions as trimers of disulfide-linked GP1-GP2 heterodimers. The GP1 subunit contains two heavily glycosylated domains, the glycan cap, and the MLD. The MLD is highly variable and contains both *N*- and *O*-linked glycans (30). The MLD is essential for immune shielding, studies have shown that the MLD-deleted GP1 and GP2 can induce an immune response that may result in cross-species immunity (102). Similarly, a subunit vaccine that contains the extracellular domain of the GP fused with the Fc fragment of human IgG1 to protect mice against EBOV lethal challenge (103). Further research found that mutation of Asn^565^ on GP2 was highly detrimental to the immunogenicity of GP; However, mutation of two *N*-glycosylation sites on GP1 (388, 415 sites) may enhance immunogenicity (62). Hence, the glycan on EBOV GP play an important role in inducing immunity, and further study of glycan may help us develop more effective vaccines.

### SARS-CoV-2

The S protein is the only antigen that is target of nAbs. The RNA vaccine that expecting S protein now aims to use all over the world. The S glycoprotein of SARS-CoV-2 is highly glycosylated with 22 *N*-glycosylation sites (104). However, in the face of mutant virus strains, existing vaccines are less effective (105, 106). Deletion of the N331 and N343 glycosylation sites of SARS-CoV-2 S protein could significantly reduce the ability of the virus to infect, and mutations of N234Q and N165Q could markedly resist to nAbs and be more sensitive, respectively (55). The functional study of different glycosylation sites on S protein may provide references for the development of effective vaccines and drugs against SARS-CoV-2 in the future.

## Application of Glycosylation Inhibitors

Glycans can affect the host cell recognition, replication, infection and immune escape of virus. With the deepening understanding of the structure and function of enveloped virus glycoproteins, research and development of antibody drugs that target enveloped virus glycoproteins have become a current hot topic (94). Studies have shown that lectins can inhibit HIV-1 infection by binding directly to the viral glycans, thereby disrupting the receptor-induced conformational changes, inhibiting membrane fusion, and blocking the binding of DC-SIGN (107–109). In addition, tetherin is a protein molecule on the surface of human cells that can block the spread of HIV and inhibit the release of a broad-spectrum of enveloped viruses by retaining virions on the surface of the infected cell. It has been reported that the antiviral activity of tetherin is related to its glycosylation. Human tetherin contains two putative *N*-linked glycosylation sites (Asn^65^ and Asn^92^), and glycosylation of at least one Asn of tetherin is necessary and sufficient for the inhibition of HIV-1 release (110). The use of mannosidase-I inhibitor such as kifunensine inhibits the biosynthesis of *N*-glycan and enhances proteolysis of S proteins, which reduces receptor-binding domain presentation on SARS-CoV-2 pseudovirus, lowers the binding to host ACE2 and decrease viral entry (111). In addition, other *N*-glycosylation inhibitors, like swainsonine, which is mannosidase II inhibitor and has been shown to be safe in humans, can cause *N*-glycan truncation may be used to reduce viral entry (112). Iminosugars are known inhibitors of αGI and αGII, like celgosivir, castanospermine, and UV-4, which can effectively inhibit the replication of SARS-CoV-2 in cell culture (113). These drugs and compounds may be used to reduce viral load and moderate SARS-CoV-2 related respiratory symptoms. Other potential inhibitors that may modulate viral entry include carbohydrate-based small molecules (e.g. 4F-GlcNAC, 4F-GalNAC) and acceptor decoys (e.g. ONAP, SNAP) are used by interfering with the mutual recognition process of glycan and lectin (114).

## Conclusions and Perspectives

**Figure 4:**
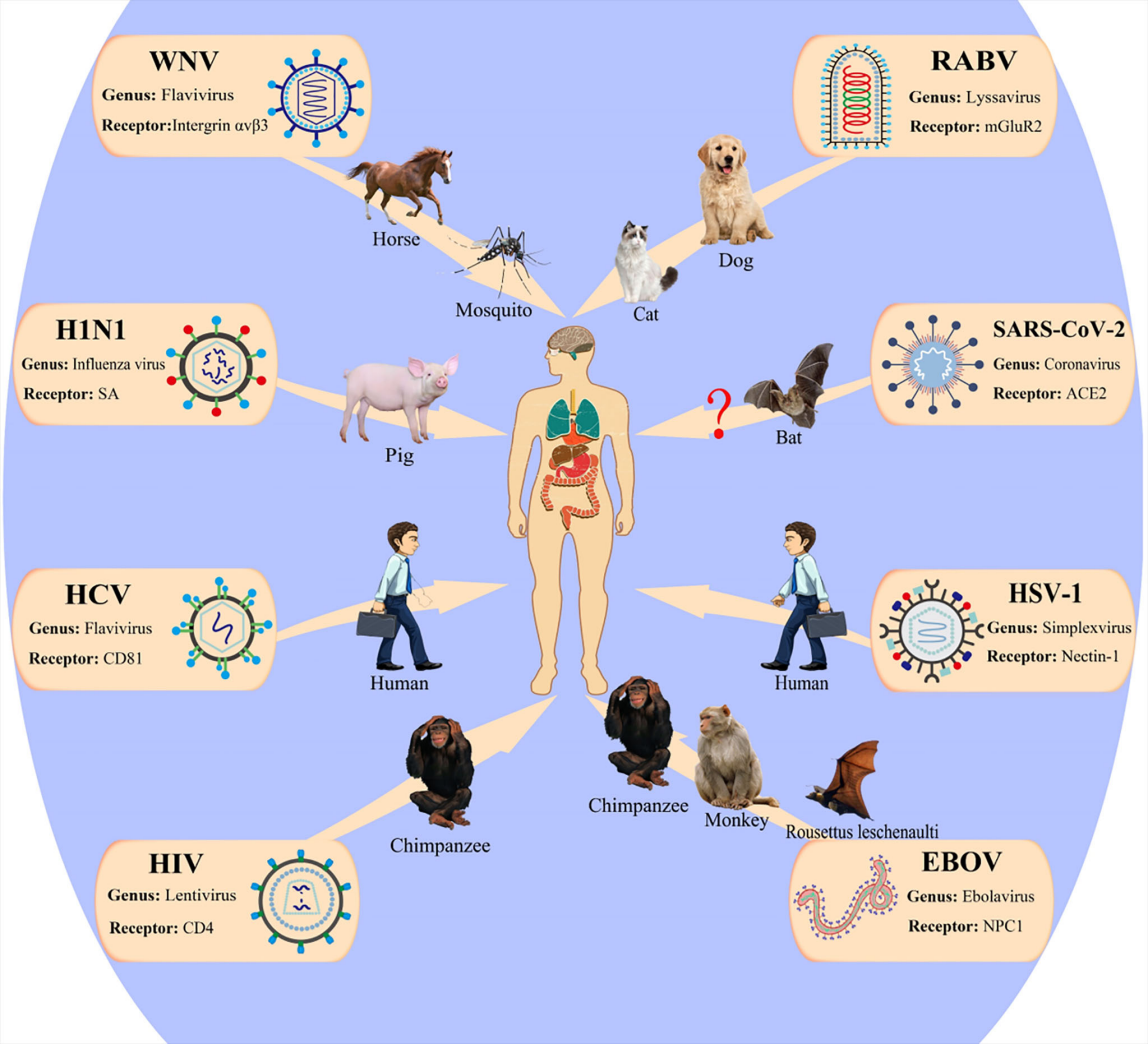
Patterns of viruses infect the human host. WNV, West Nile virus; HCV, hepatitis C virus; RABV, rabies virus; mGluR2, metabotropic glutamate receptor 2; ACE2, angiotensin-converting enzyme 2; EBOV, Ebola virus; NPC, Niemann–Pick type C; HSV, herpes simplex viruses; HIV-1, human immunodeficiency virus 1. This figure is adapted from reference (115).

Historically, viral diseases have repeatedly caused large-scale global public health concerns and threats to human health and survival. **Figure 4** illustrates the transmission pathway of several common viruses that infect humans. Increasing evidence shows that the alterations in the *N*-glycan profile and sugar recognition pattern in host cells can reflect the progress of viral infection to some extent and are expected to be a new target for the diagnosis and treatment of viral infection (116). In short, glycosylation can be a tool for the virus to infect the host and escape host immunity. Here, we have summarized the progress in studying the effects of glycan on viral behavior in recent decades, which will provide new insights for the development of viral vaccines and help to develop new targets to protect against these viruses.

Finally, faced with the ongoing COVID-19 pandemic, we need to identify the key therapeutic targets including glycosylation sites in vaccines and drug targets. With the development of the SARS-CoV-2 vaccine, although we have effective countermeasures, the mutated version of the virus still threatens the health safety of mankind. In general, the existing vaccines are still effective against the mutated virus, but the neutralization efficiency is lower (106). How to develop a more effective vaccine has become an urgent task at present. As one of the most important post-translational modifications, glycosylation is an indispensable factor in virus function. Glycosylation inhibitors can significantly inhibit viral infection and reduce the synthesis of viral proteins (117). We need to design a new vaccine virus by researching the glycosylation sites that have an impact on the viability of the virus, and modifying the glycosylation of the virus (118). Similarly, it is also very important to study SARS-CoV-2 S glycans differ from typical host glycan processing and develop targeted glycosylation inhibitors. In addition, the use of this inhibitor in combination with other types of antiviral drugs may have a better effect in combating viral infection, replication and overcoming viral resistance (119).

## Author Contributions

YL and DL wrote the manuscript. YW and WS provided language help and writing assistance. WD and GL conceived ideas and modified the manuscript. All authors contributed to the article and approved the submitted version.

## Funding

This work was supported by National Natural Science Foundation of China Research Grant (31770859), Scientific Research Foundation of Liaoning Provincial Education Department (507124), and Liaoning Provincial Program for Top Discipline of Basic Medical Sciences.

## Conflict of Interest

The authors declare that the research was conducted in the absence of any commercial or financial relationships that could be construed as a potential conflict of interest.
